# On Diphtheria

**Published:** 1879-02

**Authors:** 


On Diphtheria.—Dr. E. M. Snow says, in his last report as Registrar of the City of Providence:

In connection with this subject I think it my duty to ask the attention of the people of Providence, and especially of parents, to the following statements:

1. No case of diphtheria occurs without an adequate cause. This is self-evident.

2. The cause of nearly all cases of the disease exists in the houses or premises, or within a few feet of the houses where the same occur.

3. The cause of nearly all the cases that occur in the city is breathing impure air from privy vaults or sink drains, or cesspools; or drinking impure water.

Much observation and long-continued and careful investigation have perfectly satisfied me of the truth of these propositions, and they are applicable to all cases, whether in the tenements of the poor or in the mansions of the rich.—*Med. and Surg. Reporter.*

